# Comparison of posterior decompression techniques and conventional laminectomy for lumbar spinal stenosis

**DOI:** 10.3389/fsurg.2022.997973

**Published:** 2022-10-04

**Authors:** Yong Zhang, Fei-Long Wei, Zhi-Xin Liu, Cheng-Pei Zhou, Ming-Rui Du, Jian Quan, Yan-Peng Wang

**Affiliations:** ^1^ Department of Orthopedics, An Kang Central Hospital, AnKang, China; ^2^ Department of Orthopedics, Tangdu Hospital, Fourth Military Medical University, Xi’an, China; ^3^ Second Department of Orthopedics, Shaanxi Provincial Hospital of Traditional Chinese Medicine, Xi’an, China

**Keywords:** surgery, posterior decompression, laminectomy, lumbar spinal stenosis, outcome

## Abstract

**Objectives:**

To compare the efficacy of posterior decompression techniques with conventional laminectomy for lumbar spinal stenosis.

**Methods:**

The Embase, PubMed, and Cochrane Library databases were searched with no language limitations from inception to January 13, 2022. The main outcomes were functional disability, perceived recovery, leg and back pain, complications. A random effects model was used to pooled data. Risk ratio (RR), mean difference (MD) and 95% confidence interval (CI) were used to report results. The study protocol was published in PROSPERO (CRD42022302218).

**Results:**

14 trials including 1,106 participants were included in the final analysis. Bilateral laminotomy was significantly more efficacious in improve functionality than laminectomy [MD: −2.94; (95% CI, −4.12 to −1.76)]. Low incidence of iatrogenic instability due to bilateral laminectomy compared with laminectomy [RR: 0.11; (95% CI, 0.02 to 0.59)]. In addition, between those who received bilateral laminotomy and those undergoing laminectomy, the result showed significant difference regarding recovery [RR: 1.31; (95% CI, 1.03 to 1.67)].

**Conclusions:**

This study provides evidence that bilateral laminotomy has advantages in functional recovery, postoperative stability, and postoperative rehabilitation outcomes. Further research is needed to determine whether posterior techniques provide a safe and effective option for conventional laminectomy.

## Introduction

Lumbar spinal stenosis (LSS) refers to degenerative changes in the intervertebral discs, ligamentum flavum, and facet joints with age, resulting in narrowing of the spaces around the neurovascular structures of the spine (1). The main symptoms are leg pain, and numbness, which increases with exertion (nervous claudication) (2). When conservative treatment fails, surgical treatment should be considered. The current gold standard treatment for LSS is facet-preserving laminectomy (3). In 2007, 37,598 operations were performed due to LSS, and the total hospital cost of these operations was close to $1.65 billion (£1.1 billion; €1.55 billion) (4). Therefore, it brings a great burden to patients and society.

Conventional laminectomy requires a mid-lumbar incision to separate the paraspinous muscles from the spinous process and vertebral arch. Wide resection of bone, ligament, and muscle structures results in increased postoperative pain, blood loss, complications, and length of hospital stay (5, 6). Recently, a number of experts have recommended surgical techniques that preserve posterior midline structures (5, 7, 8). Removal of midline structures may lead to postoperative spinal instability (9). Even if the facet joints are not severely damaged, severe damage to the paraspinal muscles can lead to low back pain, which can lead to poor function (10, 11).

The microsurgical approach is ideal for adequate bilateral spinal canal or foraminal decompression with minimal separation of the paraspinal muscles (7). In particular, unilateral (6) and bilateral laminotomy (5) is used for bilateral decompression of the spinal canal. They help stabilize the spine while keeping vital bones and soft tissues safe (12). However, since most of the stability of the translational and rotational spine is provided by the intervertebral discs and facet joints (13, 14), midline structures may have little effect on spinal stability after conventional laminectomy resection. The actual efficacy of these techniques compared to conventional laminectomy is unclear. Therefore, we conducted this study to compare the efficacy of posterior decompression techniques limiting the extent of bone decompression or avoiding resection of posterior midline structures of the lumbar spine with conventional laminectomy in the treatment of LSS.

## Methods

### Data sources and search strategy

The Cochrane and PROSPERO databases were independently searched by two reviewers (Y.Z. and F.-L.W), to avoid duplicates. The Embase, PubMed, and Cochrane Library databases were searched with no language limitations from inception to January 13, 2022 (Supplementary Table S1). After the preliminary screening of titles or abstracts, two independent reviewers (Y.Z. and F.-L.W) will evaluate related publications. The protocol was registered on PROSPERO (CRD42022302218).

### Selection criteria and study design

The studies were screened according to the PICOS criteria (15). Publication inclusion criteria is outlined in Supplementary Table S2.

### Data extraction and outcomes

Data was extracted by two reviewers independently from the same set of publications including characteristics of investigators, surgical methods, characteristics of participants, and main results. The primary outcomes were functional disability, leg and back pain, complications. The second outcomes were recovery (good + excellent), instability, surgery time, perioperative blood loss, length of hospital stay, muscle cell injury (creatine kinase level), paraspinal muscle denervation/atrophy.

### Quality and risk-of-bias assessment

The Cochrane Collaboration’s risk-of-bias assessment tool (16, 17) was used to independently evaluate the included studies for potential bias. This part was completed independently by two authors. Disagreements between the two investigators were resolved by involving a third investigator (Y.-P. W). The overall risk of bias is obtained, which is divided into “high risk”, “low risk”, or “unclear risk”. Supplementary Table S3 showed the detailed information of the tool for assessing the risk of bias.

### Data synthesis and statistical analysis

Data were analyzed using STATA 16.0 (Stata Corp, College Station, TX, USA). Data were pooled using a random-effects model (18). Dichotomous data were calculated as relative risks (RR) with 95% confidence intervals (CI). Mean differences (MD) with 95% CI were used to weigh the effect size for continuous outcomes. A forest plot was used to perform effect size including the overall effect size and its 95% CI. The weight of enrolled studies depended on the value of events of the treatment group, events of the control group, and total sample size. *P *< 0.05 was considered statistically significant. Heterogeneity was assessed using *I*^2^ test (15). Data were considered with high heterogeneity if *I*^2^ > 50%. Publication bias was accounted for by Egger’s test, and significant publication bias was defined as *P* < 0.10.

## Results

### Systematic review and qualitative assessment

**Figure 1:**
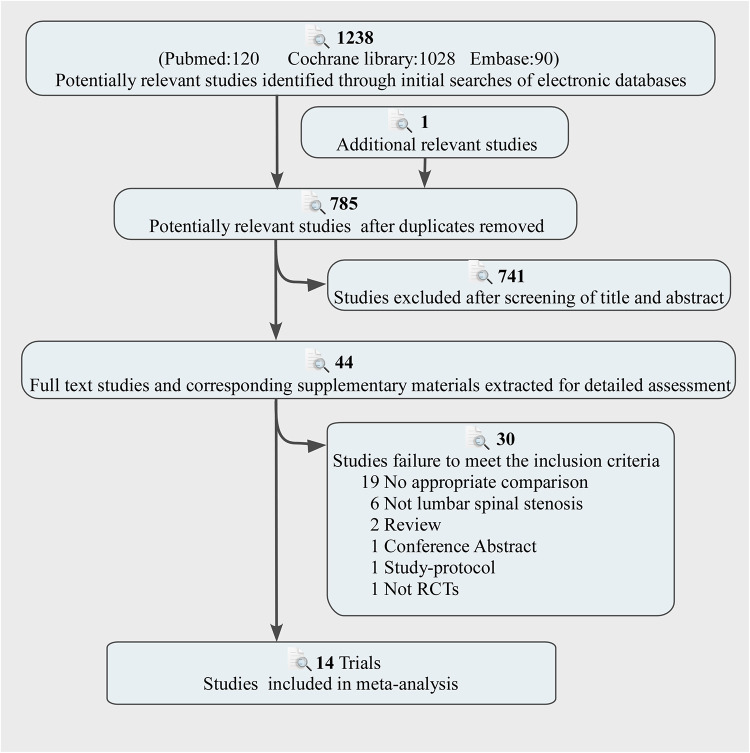
Literature search and screening process.

The flow of the selection process and the reasons for exclusion was showed in Figure 1. 1,238 publications were identified during the initial search, after excluding duplicate records (*n* = 454). Forty-four articles were retained for a comprehensive evaluation. Finally, 14 trials including 1,106 participants were included in the final analysis (5–8, 19–28). Supplementary Table S4 showed characteristics of the included trials and participants. The summary of the risk of bias assessment was displayed in Supplementary Figures S1, S2.

## Primary outcomes

### Disability

**Figure 2:**
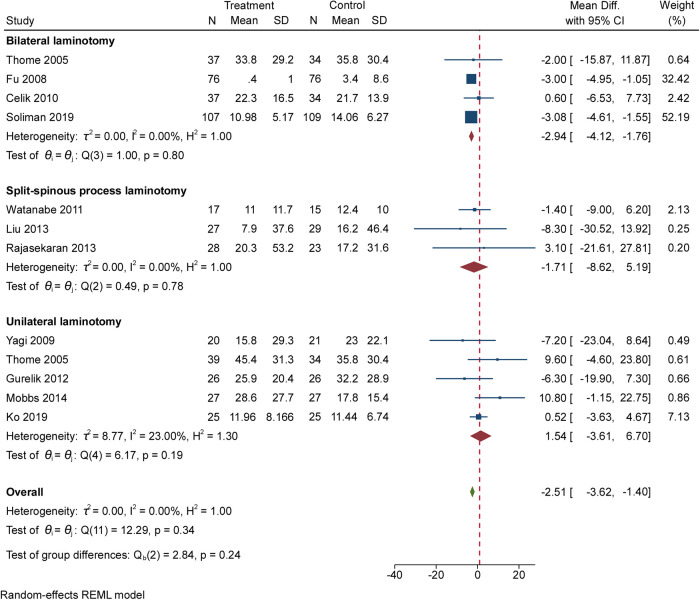
The forest plot regarding standardized disability index compared posterior techniques with tourniquet group.

Eleven RCTs (919 participants) compared the differences in disability under different interventions (Figure 2) (5–7, 20–25, 27, 28). No valid disability score was reported by Postacchini et al. (8). The result showed no significant difference in disability scores between patients undergoing unilateral laminectomy and laminectomy [5 RCTs, 277 participants, MD: 1.54; (95% CI, −3.61 to 6.70)]. Between those who received bilateral laminotomy and those undergoing laminectomy, the result showed a significant difference regarding disability scores in favour of bilateral laminotomy [4 RCTs, 510 participants, MD: −2.94; (95% CI, −4.12 to −1.76)]. Between those who received split-spinous process laminotomy and those undergoing laminectomy, the result showed no significant difference regarding disability scores [3 RCTs, 139 participants, MD: −1.71; (95% CI, −8.62 to 5.19)]. Low heterogeneity was found across studies reporting disability. Egger’s test (*P* = 0.9372, 0.4524 and 0.8921, respectively) revealed no significant publication bias in disability.

### Leg pain

**Figure 3:**
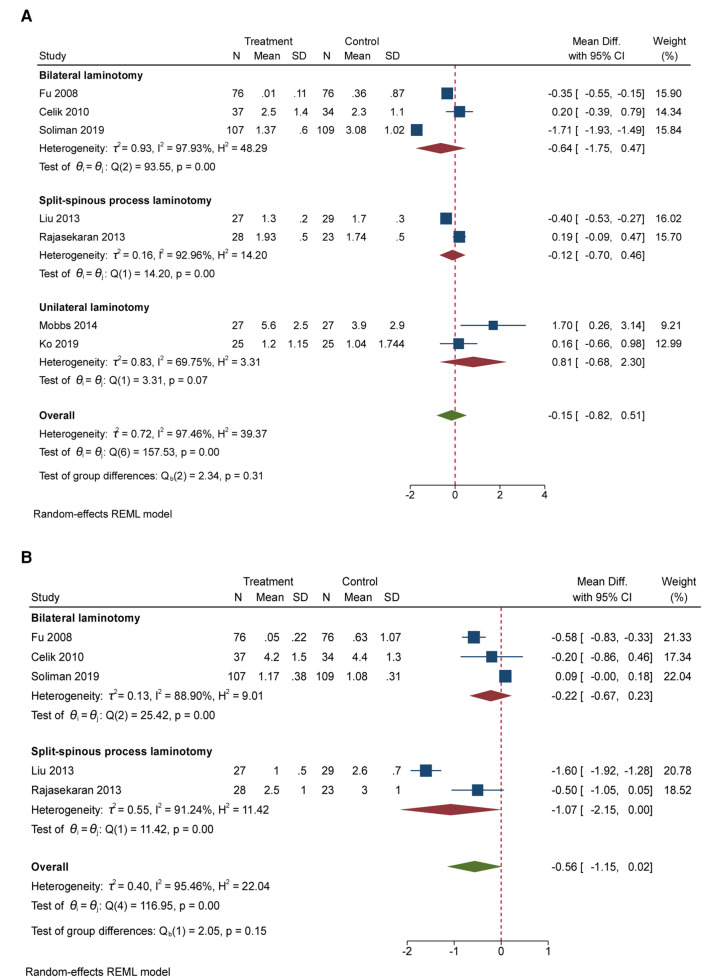
(**A**) The forest plot regarding leg pain compared posterior techniques with tourniquet group. (**B**) The forest plot regarding back pain compared posterior techniques with tourniquet group.

Seven RCTs (650 participants) compared the differences in leg pain under different interventions (Figure 3A) (5, 7, 20, 24, 25, 27, 28). No valid pain score was reported by Postacchini et al. (8). The result showed no significant difference in leg pain between patients undergoing unilateral laminectomy and laminectomy [2 RCTs, 104 participants, MD: 0.81; (95% CI, −0.68 to 2.30)]. Between those who received bilateral laminotomy and those undergoing laminectomy, the result showed no significant difference regarding leg pain [3 RCTs, 439 participants, MD: −0.64; (95% CI, −1.75 to 0.47)]. Between those who received split-spinous process laminotomy and those undergoing laminectomy, the result showed no significant difference regarding leg pain [2 RCTs, 107 participants, MD: −0.12; (95% CI, −0.70 to 0.46)]. High heterogeneity was found across studies reporting leg pain. Egger’s test (*P* = 0.3686) revealed no significant publication bias in leg pain between bilateral laminotomy and laminectomy.

### Back pain

Five RCTs (546 participants) compared the differences in back pain under different interventions (Figure 3B) (5, 7, 20, 24, 25). Between those who received bilateral laminotomy and those undergoing laminectomy, the result showed no significant difference regarding back pain [3 RCTs, 439 participants, MD: −0.22; (95% CI, −0.67 to 0.23)]. Between those who received split-spinous process laminotomy and those undergoing laminectomy, the result showed significant difference regarding back pain [2 RCTs, 107 participants, MD: −1.07; (95% CI, −2.15 to 0.00)]. High heterogeneity was found across studies reporting back pain. Egger’s test (*P* = 0.7974) revealed no significant publication bias in back pain between bilateral laminotomy and laminectomy.

### Complications

**Figure 4:**
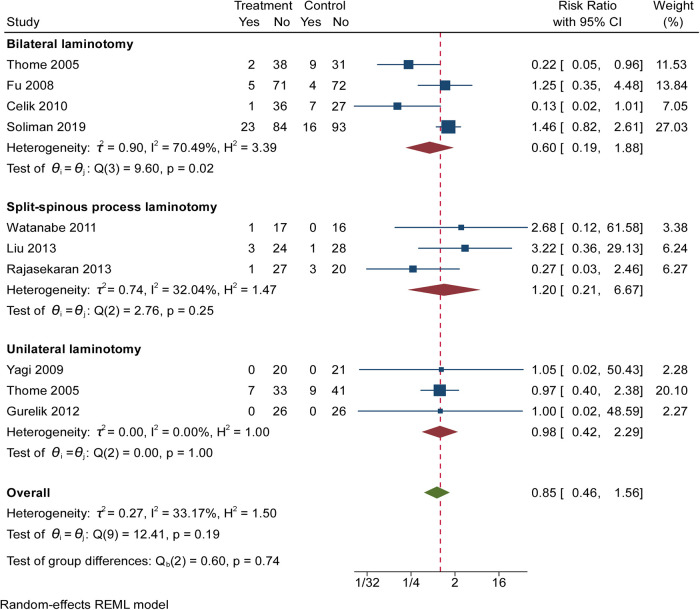
The forest plot regarding complications compared posterior techniques with tourniquet group.

Nine RCTs (843 participants) compared the differences in complications under different interventions (Figure 4) (5–7, 20–25). The result showed no significant difference in complications rates between patients undergoing unilateral laminectomy and laminectomy [3 RCTs, 183 participants, RR: 0.98; (95% CI, 0.42 to 2.29)]. Between those who received bilateral laminotomy and those undergoing laminectomy, the result showed no significant difference regarding complications rates [4 RCTs, 519 participants, RR: 0.60; (95% CI, 0.19 to 1.88)]. Between those who received split-spinous process laminotomy and those undergoing laminectomy, the result showed no significant difference regarding complications rates [3 RCTs, 141 participants, RR: 1.20; (95% CI, 0.21 to 6.67)]. High heterogeneity was found across studies reporting complications between bilateral laminotomy and laminectomy. Egger’s test (*P* = 0.0254) revealed significant publication bias in complications between bilateral laminotomy and laminectomy. Low heterogeneity was found across studies reporting complications between unilateral laminotomy, split-spinous process laminotomy and laminectomy. Egger’s test (*P* = 0.9721 and 0.6580, respectively) revealed no significant publication bias.

## Second outcomes

### Instability

**Figure 5:**
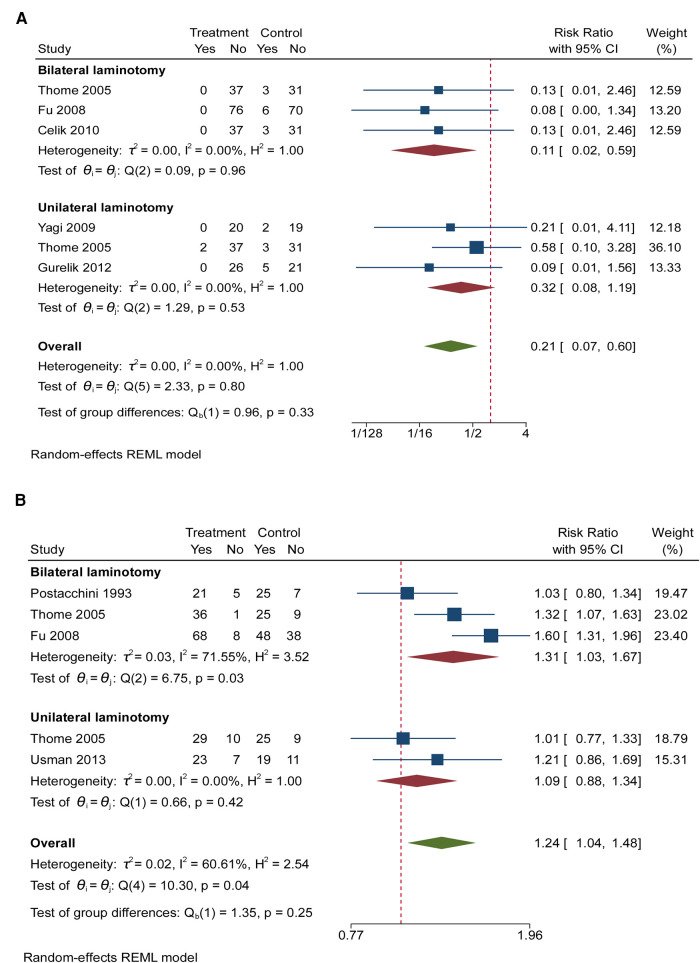
(**A**) The forest plot regarding instability compared posterior techniques with tourniquet group. (**B**) The forest plot regarding recovery (good + excellent) compared posterior techniques with tourniquet group.

Five RCTs (460 participants) compared the differences in instability under different interventions (Figure 5A) (5, 6, 20, 21, 23). Between those who received unilateral laminotomy and those undergoing laminectomy, the result did not show significant difference regarding instability [3 RCTs, 166 participants, RR: 0.32; (95% CI, 0.08 to 1.19)]. Between those who received bilateral laminotomy and those undergoing laminectomy, the result showed significant difference regarding instability [3 RCTs, 294 participants, RR: 0.11; (95% CI, 0.02 to 0.59)]. Low heterogeneity was found across studies reporting instability. Egger’s test (*P* = 0.7656 and 0.3026, respectively) revealed no significant publication bias in instability.

### Recovery (good + excellent)

Four RCTs (460 participants) compared the differences in recovery under different interventions (Figure 5B) (6, 8, 20, 26). Between those who received unilateral laminotomy and those undergoing laminectomy, the result did not show significant difference regarding disability scores [3 RCTs, 166 participants, RR: 1.09; (95% CI, 0.88 to 1.34)]. Between those who received bilateral laminotomy and those undergoing laminectomy, the result showed significant difference regarding recovery [3 RCTs, 291 participants, RR: 1.31; (95% CI, 1.03 to 1.67)]. Low heterogeneity was found across studies reporting recovery between unilateral laminotomy and laminectomy. But Egger’s test (*P* = 0.0233) revealed significant publication bias.

### Length of surgical procedure

**Figure 6:**
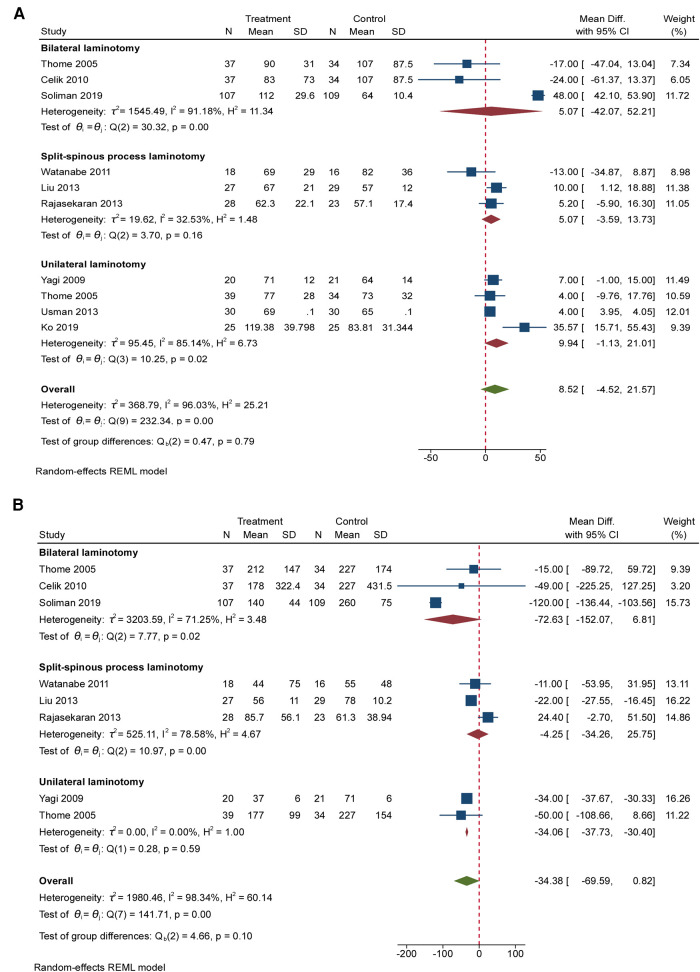
(**A**) The forest plot regarding instability compared posterior techniques with tourniquet group. (**B**) The forest plot regarding recovery (good + excellent) compared posterior techniques with tourniquet group.

Ten RCTs (793 participants) compared the differences in length of surgical procedure under different interventions (Figure 6A) (5–7, 19, 21, 22, 24–26, 28). The result showed no significant difference in length of surgical procedure between patients undergoing unilateral laminectomy and laminectomy [4 RCTs, 224 participants, MD: 9.94; (95% CI, −1.13 to 21.01)]. Between those who received bilateral laminotomy and those undergoing laminectomy, the result showed no significant difference regarding length of surgical procedure [3 RCTs, 358 participants, MD: 5.07; (95% CI, −42.07 to 52.21)]. Between those who received split-spinous process laminotomy and those undergoing laminectomy, the result showed no significant difference regarding length of surgical procedure [4 RCTs, 211 participants, MD: 5.07; (95% CI, −3.59 to 13.73)]. High heterogeneity was found across studies reporting length of surgical procedure between unilateral laminectomy, bilateral laminotomy and laminectomy. Low heterogeneity was found across studies reporting length of surgical procedure between split-spinous process laminotomy and laminectomy. Egger’s test (*P* = 0.000) revealed significant publication bias in complications between bilateral laminotomy and laminectomy. Egger’s test (*P* = 0.1216 and 0.1511, respectively) revealed no significant publication bias between unilateral laminectomy, split-spinous process laminotomy and laminectomy.

### Blood loss

Eight RCTs (683 participants) compared the differences in blood loss under different interventions (Figure 6B) (5–7, 19, 21, 22, 24, 25). The result showed significant difference in blood loss between patients undergoing unilateral laminectomy and laminectomy [2 RCTs, 114 participants, MD: −34.06; (95% CI, −37.73 to 30.40)]. Between those who received bilateral laminotomy and those undergoing laminectomy, the result showed no significant difference regarding blood loss [3 RCTs, 358 participants, MD: −72.63; (95% CI, −152.07 to 6.81)]. Between those who received split-spinous process laminotomy and those undergoing laminectomy, the result showed no significant difference regarding blood loss [4 RCTs, 211 participants, MD: −4.25; (95% CI, −34.26 to 25.75)]. High heterogeneity was found across studies reporting complications between bilateral laminotomy, split-spinous process laminotomy and laminectomy. Low heterogeneity was found across studies reporting blood loss between unilateral laminectomy and laminectomy. Egger’s test (*P* = 0.3670 and 0.3198, respectively) revealed no significant publication bias between bilateral laminotomy, split-spinous process laminotomy and laminectomy.

## Discussion

LSS is more common in the elderly over 60 years old (29), mainly causing radiating calf pain and intermittent neurogenic claudication (28). The primary surgical treatment for LSS is adequate decompression. Our previous study found an increased complication rate for combined decompression and fusion surgery, and there is no evidence that it is superior to decompression alone (30). Existing decompression alone surgery mainly includes conventional laminectomy, unilateral laminectomy, bilateral laminotomy and split-spinous process laminotomy. However, it is unclear whether these posterior approaches are superior to conventional laminectomy. So we conducted this meta-analysis included 14 trials including 1,106 participants. Ultimately, we concluded that bilateral laminectomy was superior to conventional laminectomy.

Functional impairment, sensory recovery, and leg pain are the most important aspects of LSS to guide decisions about specific techniques. In our study, the success rate (good + excellent) of conventional laminectomy was only 65.7% lower than that of the posterior approaches of 85.1%. But the pooled results showed that only bilateral laminotomy reduces the risk of instability which was consistent with previous study (31). And unilateral laminectomy or spinous split laminectomy did not show a particular benefit in improving function and reducing pain compared with conventional laminectomy.

When there is no significant difference in the primary outcomes among several surgical methods, the secondary outcomes can provide a key guidance for choosing the appropriate surgery. One of the benefits of conventional laminectomy is that it provides good visibility and adequate working space. However, secondary spinal instability may result due to the removal of too much posterior structure. In our included RCTs, posterior laminectomy reduced the risk of instability compared with conventional laminectomy. But the pooled results showed that only bilateral laminotomy reduces the risk of instability which was consistent with previous study (31). Postoperative instability is considered an important cause of low back pain (32). Instability is also an important cause of revision surgery (33). Because of the lack of a clear definition of spinal instability (34), the true incidence of postoperative instability in lumbar spinal stenosis is unknown. In the studies we included, the rate of instability with conventional laminectomy was 9.8% (5, 6, 20, 21, 23). Only three studies reported the incidence of reoperation due to vertebral instability (5, 6, 20). And the length of the follow-up period and thus the likelihood of developing instability varies widely between studies. Therefore, further research is needed on the relationship between decompression techniques and the incidence of instability.

Compared with laminectomy, the technique of preserving the posterior midline structure may result in increased surgical length due to limited operating space. But our study did not find statistical significance, which is inconsistent with some studies (7, 28). This may be related to the technical proficiency of the operator. Unilateral laminectomy results in significantly less blood loss than conventional laminectomy due to less damage to the posterior structures of the spine (6, 21). In addition, the rate of muscle atrophy ratio of paravertebral was significantly lower with split-spinous process laminotomy compared to laminectomy (Supplementary Figure S3). But the results showed no significant differences regarding postoperative creatine kinase levels (Supplementary Figure S4). Furthermore, our study showed no statistical difference between the posterior technique and laminectomy in terms of length of hospital stay (Supplementary Figure S5).

## Limitations

The advantages of bilateral laminectomy for iatrogenic instability are credible, but final conclusions are limited by methodological differences and the low quality of included studies. Inconsistencies in outcome measures and follow-up time points contribute to heterogeneity of results, and standardization of outcome measures and follow-up time points can improve comparability of studies. Furthermore, long-term efficacy assessments of these techniques are currently lacking. Therefore, more rigorous methodological studies are needed to compare techniques for decompression of lumbar spinal stenosis.

## Conclusions

This study provides evidence that bilateral laminotomy has advantages in functional recovery, postoperative stability, and postoperative rehabilitation outcomes. Further research is needed to determine whether posterior techniques provide a safe and effective option for conventional laminectomy.

## Data Availability

The original contributions presented in the study are included in the article/**Supplementary Material**, further inquiries can be directed to the corresponding author/s.
