# Supplementary material for: Comparison of posterior decompression techniques and conventional laminectomy for lumbar spinal stenosis
Source: Front Surg. 2022 Oct 4;9:997973. doi: 10.3389/fsurg.2022.997973 (PMC9577104; doi:10.3389/fsurg.2022.997973)
Supplement: Supplementary file 1 [file DataSheet1.docx]

**Supplementary files**

**1. Figures** (Supplementary Figure 1- Supplementary Figure 49)

**Supplementary Figure 1.** Risk of bias summary of included studies.

**Supplementary Figure 2.** Risk of bias of included studies.

**Supplementary Figure 3.** Forest plot of comparison: split-spinous process laminotomy compared with conventional laminectomy regarding muscle atrophy ratio of paravertebral muscle.

**Supplementary Figure 4.** Forest plot of comparison: split-spinous process laminotomy compared with conventional laminectomy regarding muscle cell injury (creatine kinase level IU/L).

**Supplementary Figure 5.** Forest plot of comparison: posterior technique compared with conventional laminectomy regarding muscle atrophy ratio of paravertebral muscle.

**2. Tables** (Supplementary Table 1- Supplementary Table 19)

**Supplementary Table 1.** Search strategy.

**Supplementary Table 2.** Inclusion/exclusion criteria of literature.

**Supplementary Table 3.** Risk of bias table.

**Supplementary Table 4.** Characteristics of the Included Trials and Participants.

**Supplementary Figure 1. Risk of bias summary of included studies.**


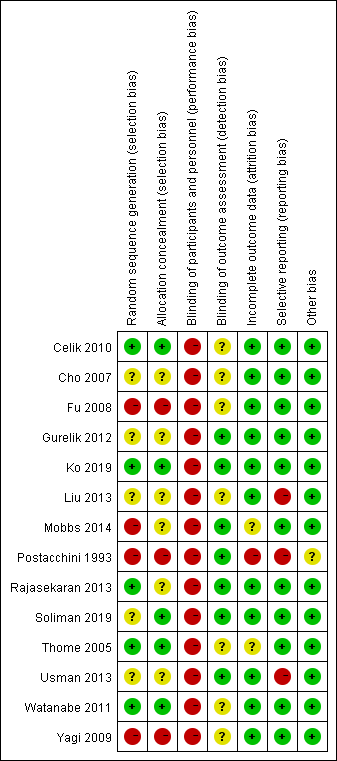


**Supplementary Figure 2.** **Risk of bias of included studies.**


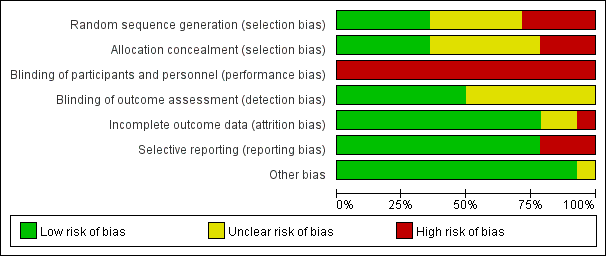


**Supplementary Figure 3.** **Forest plot of comparison: split-spinous process laminotomy compared with conventional laminectomy regarding muscle atrophy ratio of paravertebral muscle.**


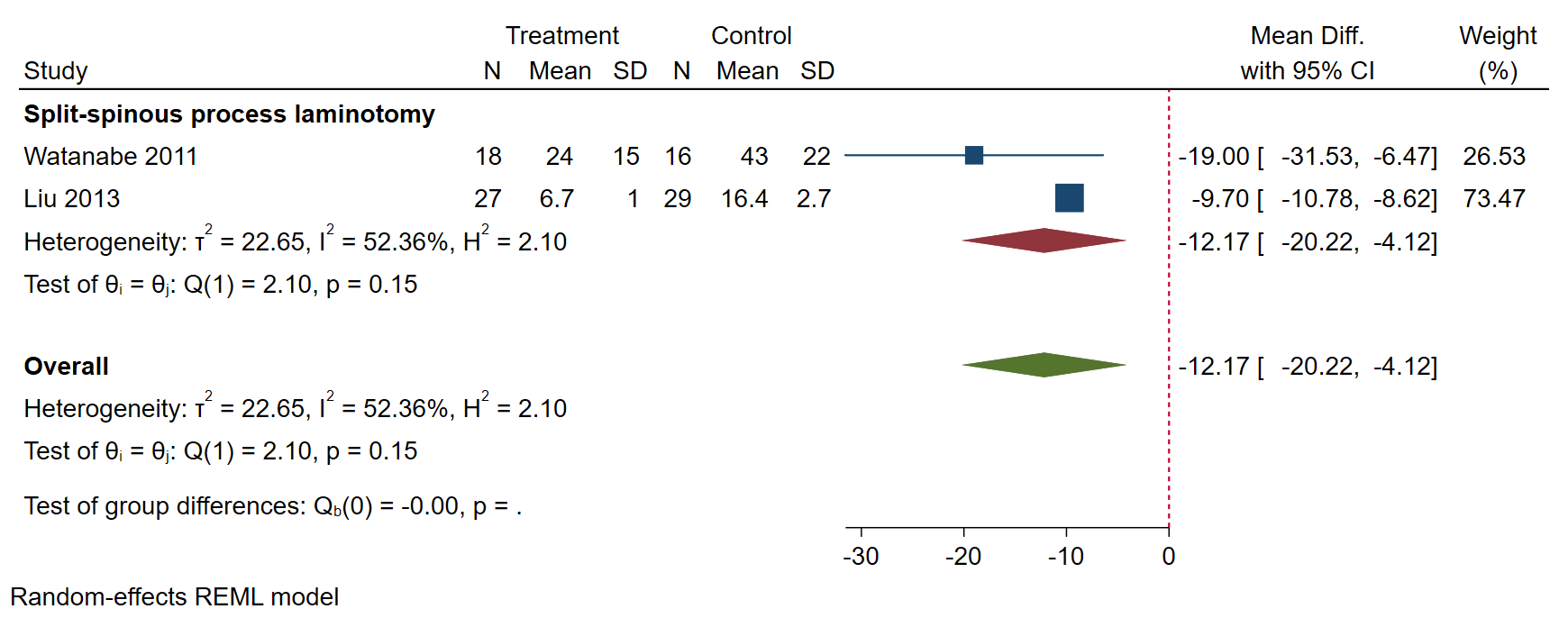


**Supplementary Figure 4.** **Forest plot of comparison: split-spinous process laminotomy compared with conventional laminectomy regarding muscle cell injury (creatine kinase level IU/L).**
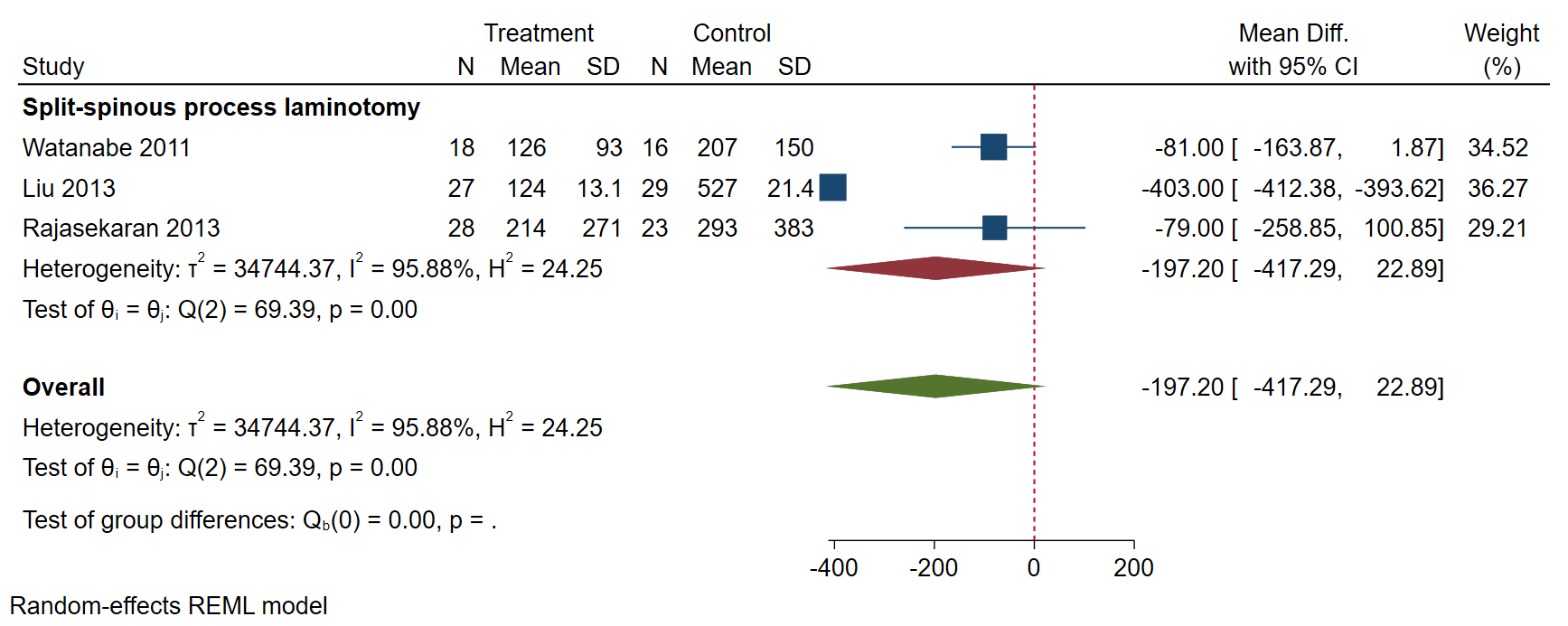


**Supplementary Figure 5.** **Forest plot of comparison: posterior technique compared with conventional laminectomy regarding muscle atrophy ratio of paravertebral muscle.**


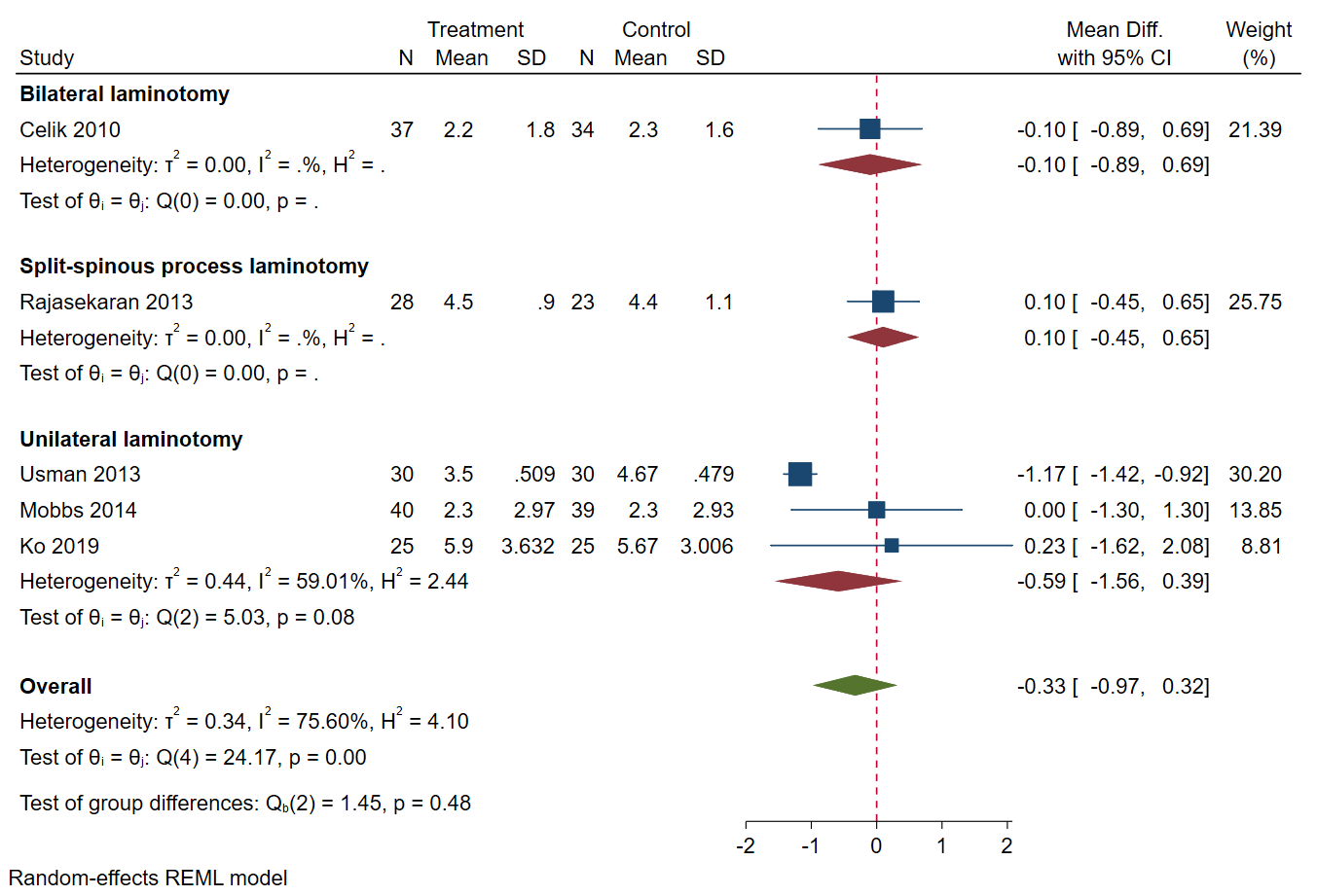


**Supplementary Table 1. Search strategy**

| Database | Search strategy |
| --- | --- |

| Pubmed | #1 ((low back) OR (lumbar)) OR (canal)  #2 (stenos) OR (Spinal Stenosis[MeSH Terms])  #3 #1 AND #2  #4 (((((((((((((Decompression, Surgical[MeSH Terms]) OR (laminectomy[MeSH Terms])) OR (Surgical Procedures, Minimally Invasive[MeSH Terms])) OR (osteotomy[MeSH Terms])) OR (Endoscopy[MeSH Terms])) ) OR (decompress)) OR (laminectom)) OR (laminotom)) OR (osteotom)) OR (enlargement)) OR (minimally invasive)) OR (endoscop)  #5 #3 and #4  #6 #5 AND randomized controlled trial (pub) |
| --- | --- |
| Embase | #1 ((low back) OR (lumbar)) OR (canal)  #2 (stenos*.mp) AND (vertebral canal stenosis)  #3 #1 AND #2  #4 (decompression surgery) OR (nerve decompression) OR (laminectomy) OR (minimally invasive surgery) OR (osteotomy) OR (endoscopic surgery) OR (decompress*.mp) OR (laminectom*.mp) OR (laminotom*.mp) OR (osteotom*.mp) OR (enlargement.mp) OR (minimally invasive.mp) OR (endoscop*.mp)  #5 #3 AND #4  #6 #5 AND [randomized controlled trial]/lim |
| Cochrane library | #1 ((low back) OR (lumbar)) OR (canal)  #2 (stenos) OR MeSH descriptor: [Spinal Stenosis] explode all trees  #3 #1 AND #2  #4 MeSH descriptor: [Decompression, Surgical] this term only AND MeSH descriptor: [Laminectomy] this term only AND MeSH descriptor: [Surgical Procedures, Minimally Invasive] this term only AND MeSH descriptor: [Osteotomy] this term only AND MeSH descriptor: [Endoscopy] this term only AND decompress AND laminectomy AND laminotom AND osteotom AND enlargement AND minimally invasive AND endoscop  #5 #3 AND #4  #6 #5 in Trials |

**Supplementary Table 2. Inclusion/exclusion criteria of literature**

| **PICOS** | **Inclusion** | **Exclusion** |
| --- | --- | --- |
| P | Patients with symptomatic degenerative lumbar stenosis. | Cases of congenital lumbar stenosis (e.g. achondroplasia) or acquired lumbar stenosis due to trauma, infection or abnormal bone metabolism (e.g. Paget's disease). |
| I | 1) posterior decompressive technique that avoids removal of posterior midline structures (spinous processes, vertebral arches, interspinous and supraspinous ligaments) or a technique involving only partial resection of the vertebral arch;  2) No limit on sample size. | removal of posterior midline structures (spinous processes, vertebral arches, interspinous and supraspinous ligaments). |
| C | Conventional facet-preserving laminectomy. | Cases of decompression through interspinous process devices or concomitant (instrumented) fusion procedures. |
| O | 1) Primary outcomes including functional disability (e.g. Roland Disability Questionnaire,  Oswestry Disability Index), perceived recovery, leg and back pain, complications.  2) Secondary outcome included length of hospital stay, recovery (good + excellent), instability, surgery time, perioperative blood loss, muscle cell injury (creatine kinase level), paraspinal muscle denervation/atrophy. | Relevant outcomes were missing. |
| S | RCT irrespective of blinding or arm. | 1) Articles without peer-reviewed or unpublished;  2) Studies that were repeatedly published or had qualitative outcomes;  3) Quasi-experimental studies, crossover, and observational studies. |

We included all prospective studies comparing a posterior

decompressive technique that avoids removal of posterior midline

structures (spinous processes, vertebral arches, interspinous and

supraspinous ligaments) or a technique involving only partial

resection of the vertebral arch with conventional facet-preserving

laminectomy. We also included studies that describe cases

requiring decompression of more than one stenotic level or a

concomitant discectomy or foraminotomy.

We excluded studies involving cases of decompression through

interspinous process devices or concomitant (instrumented) fusion

procedures.

**Supplementary Table 3. Risk of bias table.**

| 1 | Random sequence generation (selection bias) |
| --- | --- |
| 2 | Allocation concealment (selection bias) |
| 3 | Blinding of participants and personnel (performance bias) |
| 4 | Blinding of outcome assessment (detection bias) |
| 5 | Incomplete outcome data (attrition bias) |
| 6 | Selective reporting (reporting bias) |
| 7 | Other bias |

**eTable 4. Characteristics of the Included Trials and Participants.**

| Number | Study | Study design | Comparison groups | Number of participants | Age, years | Male/female | Length of  follow-up | Complete  follow-up | Outcomes |
| --- | --- | --- | --- | --- | --- | --- | --- | --- | --- |
| 1 | Postacchini 1993 | RCT | 1) Bilateral laminotomy  2) Conventional laminectomy | 1) 26  2) 32 | 57 (43 to 79) | 34/ 36 | 3.7 years | 67/ 70 | Recovery, VAS leg pain (improvement), VAS back pain (improvement), operation duration, blood loss. |
| 2 | Thome 2005 | RCT | 1) Bilateral laminotomy  2) Unilateral laminotomy  3) Conventional laminectomy | 1) 37  2) 39  3) 34 | 1) 70±7  2) 67±9  3) 69±10 | 1) 20/ 20  2) 15/ 25  3) 18/ 22 | 15.5 months | 1) 37/ 39  2) 39/ 40  3) 34/ 38 | RDQ, recovery, leg pain (improvement), complications, instability, walking distance, VAS back pain (improvement), operation duration, blood loss. |
| 3 | Cho 2007 | RCT | 1) Split-spinous process  laminotomy  2) Conventional laminectomy | 1) 40  2) 30 | 1) 61±11  2) 59±15 | 1) 16/ 24  2) 15/ 15 | 1) 15.1 months 2) 14.8 months | Not specified | JOA， Length of hospital stay, complications, instability, muscle cell injury, VAS back pain, operation duration, blood loss. |
| 4 | Fu 2008 | RCT | 1) Bilateral laminotomy  2) Conventional laminectomy | 1) 76  2) 76 | 1) 57 (47to 70)  2) 57 (45 to 73) | 1) 37/ 39  2) 33/ 43 | 40.6 months | Not specified | ODI, recovery, VAS leg pain, Complications, instability, walking duration, VAS back pain. |
| 5 | Yagi 2009 | RCT | 1) Unilateral microendoscopic laminotomy  2) Conventional laminectomy | 1) 20  2) 21 | 1)73.3 (63to79)  2)70.8 (66to73) | 1) 8/ 12  2) 6/ 15 | 1) 18.8 months  2) 18.6 months | Not specified | JOA, Length of hospital stay, complications, instability, muscle atrophy, muscle cell injury, VAS back pain, operation duration, blood loss, analgesics. |
| 6 | Celik 2010 | RCT | 1) Bilateral laminotomy  2) Conventional laminectomy | 1) 37  2) 34 | 1) 59±14  2) 61±13 | 1) 17/ 20  2) 16/ 18 | 1) 5.4 years  2) 5.3 years | 1) 37 of 40 lost to follow-up  2) 34 of 40 lost to follow-up | ODI, VAS，Length of hospital stay, complications, instability, walking distance, VAS back pain, operation duration, blood loss, analgesics. |
| 7 | Watanabe  2011 | RCT | 1) Split-spinous process laminotomy  2) Conventional laminectomy | 1) 18  2) 16 | 1)69±10  2)71±8 | 1) 10/ 8 2) 8/ 8 | 1 year | 32/34 | JOA, muscle cell injury, back muscle atrophy, blood loss, operating time, analgesics, complications. |
| 8 | Gurelik  2012 | RCT | 1) Unilateral laminotomy  2) Conventional laminectomy | 1) 26  2) 26 | 1) 61±10  2) 58±9 | 1) 11/ 15  2) 10/ 16 | 9.1 months | Not specified | ODI, Complications, instability, walking distance. |
| 9 | Liu 2013 | RCT | 1) Split-spinous process with unilateral osteotomy and laminotomy  2) Conventional laminectomy | 1) 27  2) 29 | 1) 59±4.7  2) 61±3.1 | 1) 15/ 12  2) 18/ 11 | 2 years | Not specified | JOA, VAS leg pain, VAS back pain, muscle atrophy, muscle cell injury, complications, instability, operation time, blood loss. |
| 10 | Rajasekaran  2013 | RCT | 1) Split-spinous process laminotomy  2) Conventional laminectomy | 1) 28  2) 23 | 1) 57.3±11.2  2) 54.5±8.2 | 1) 16/ 12  2) 14/ 9 | 14.2 months | 51/ 52 | JOA, VAS leg pain, VAS back pain, muscle cell injury, blood loss, operating time, duration of hospital stay, complications. |
| 11 | Usman  2013 | RCT | 1) Unilateral laminotomy  2) Conventional laminectomy | 1) 30  2) 30 | 73.4% between 31-50 years | 1) 16/ 14  2) 18/ 12 | 3 months | 1) 30/ 30  2) 30/ 30 | Operation time, length of hospital stay. |
| 12 | Mobbs  2014 | RCT | 1) Conventional Laminectomy  2) Unilateral laminotomy | 1) 40  2) 39 | 1) 66.2±8.110  2) 68.1±10.716 | 1) 1:1  2) 1:5 | 24 months | 1) 27/ 40  2) 27/ 39 | ODI, RMDQ, SF-36, Pain, Operation time, hospitalization. |
| 13 | Soliman 2019 | RCT | 1) Bilateral laminotomy  2) Conventional Laminectomy | 1) 109  2) 109 | 1) 54.21±4.54  2) 52.88±4.19 | 1) 60/ 49  2) 56/ 53 | 3 years | 1) 109/ 109  2) 107/ 109 | VAS leg pain, VAS back pain, ODI, neurogenic claudication (ZCQ) scores, surgical time, blood loss. |
| 14 | Ko 2019 | RCT | 1) Unilateral laminotomy  2) Conventional Laminectomy | 1) 27  2) 27 | 1) 68.08±10.716  2) 66.24±8.110 | 1) 10/ 15  2) 8/ 17 | 24 months | 1) 25/ 27  2) 25/ 27 | ODI, RMDQ, SF-36, Pain, Operation time, hospitalization. |
