# Supplementary material for: Comparison of posterior decompression techniques and conventional laminectomy for lumbar spinal stenosis
Source: Front Surg. 2022 Oct 4;9:997973. doi: 10.3389/fsurg.2022.997973 (PMC9577104; doi:10.3389/fsurg.2022.997973)
Supplement: Supplementary file 2 [file DataSheet1.pdf]

Supplementary files

1. Figures (Supplementary Figure 1- Supplementary Figure 5)

Supplementary Figure 1. Risk of bias summary of included studies.

Supplementary Figure 2. Risk of bias of included studies.

Supplementary Figure 3. Forest plot of comparison: split-spinous process laminotomy compared with conventional laminectomy regarding muscle atrophy ratio of paravertebral muscle.

Supplementary Figure 4. Forest plot of comparison: split-spinous process laminotomy compared with conventional laminectomy regarding muscle cell injury (creatine kinase level IU/L).

Supplementary Figure 5. Forest plot of comparison: posterior technique compared with conventional laminectomy regarding muscle atrophy ratio of paravertebral muscle.

2. Tables (Supplementary Table 1- Supplementary Table 4)

Supplementary Table 1. Search strategy.

Supplementary Table 2. Inclusion/exclusion criteria of literature.

Supplementary Table 3. Risk of bias table.

Supplementary Table 4. Characteristics of the Included Trials and Participants.

Supplementary Figure 1. Risk of bias summary of included studies.

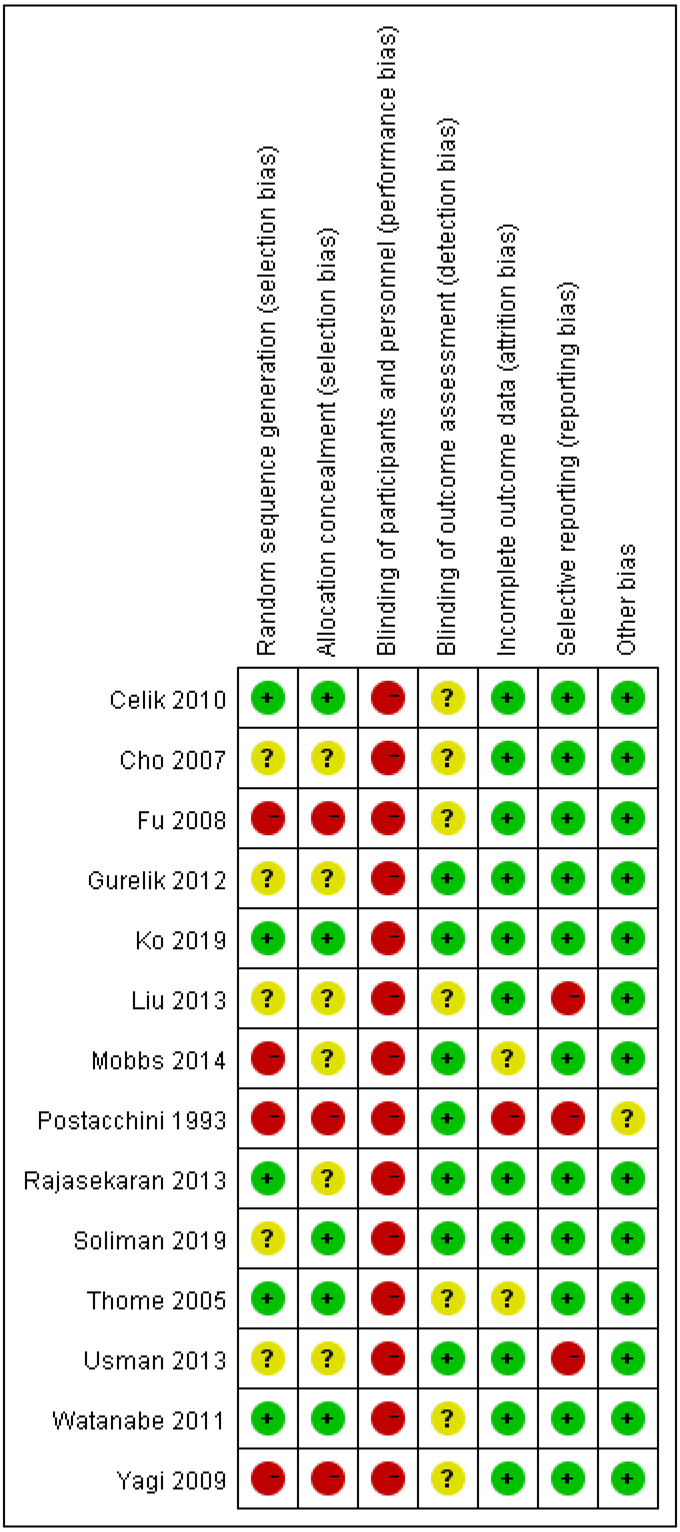

Supplementary Figure 2. Risk of bias of included studies.

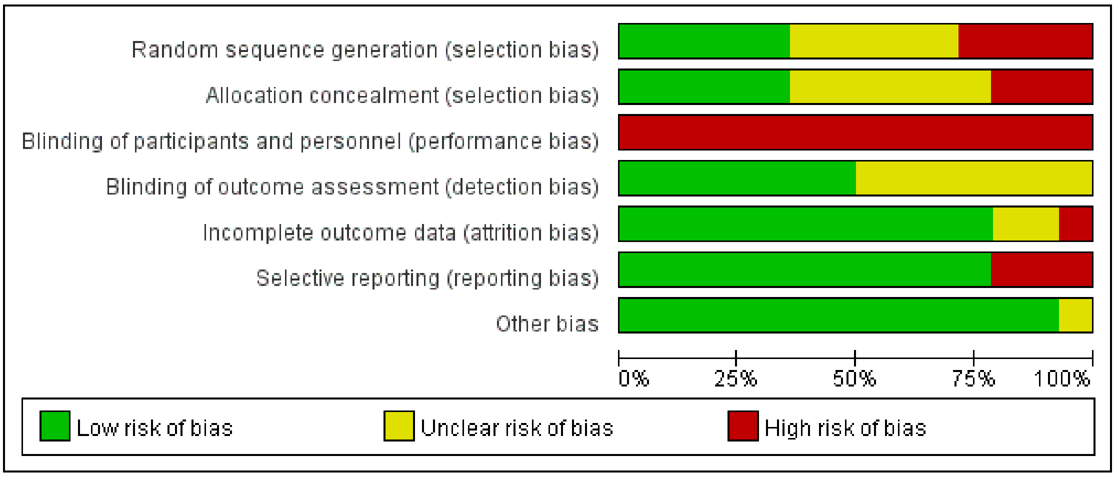

Supplementary Figure 3. Forest plot of comparison: split-spinous process laminotomy compared with conventional laminectomy regarding muscle atrophy ratio of paravertebral muscle.

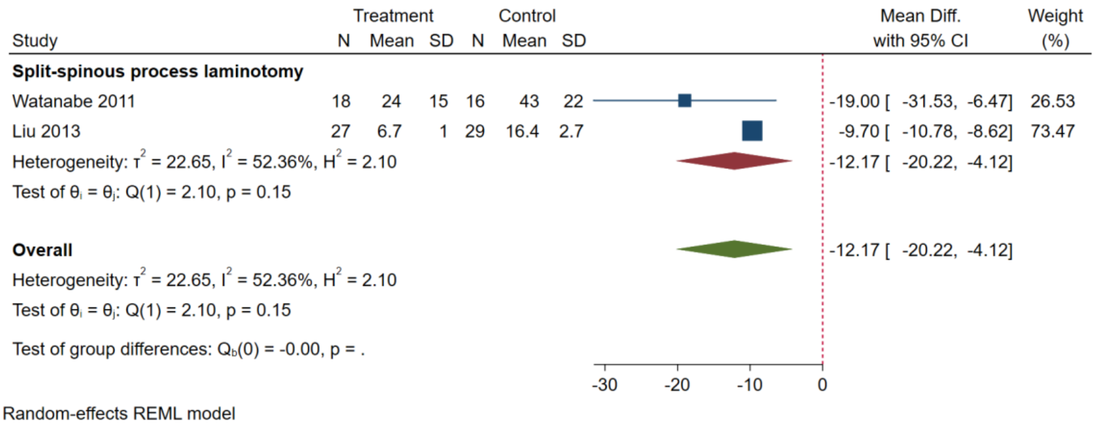

Supplementary Figure 4. Forest plot of comparison: split-spinous process laminotomy compared with conventional laminectomy regarding muscle cell injury (creatine kinase level IU/L).

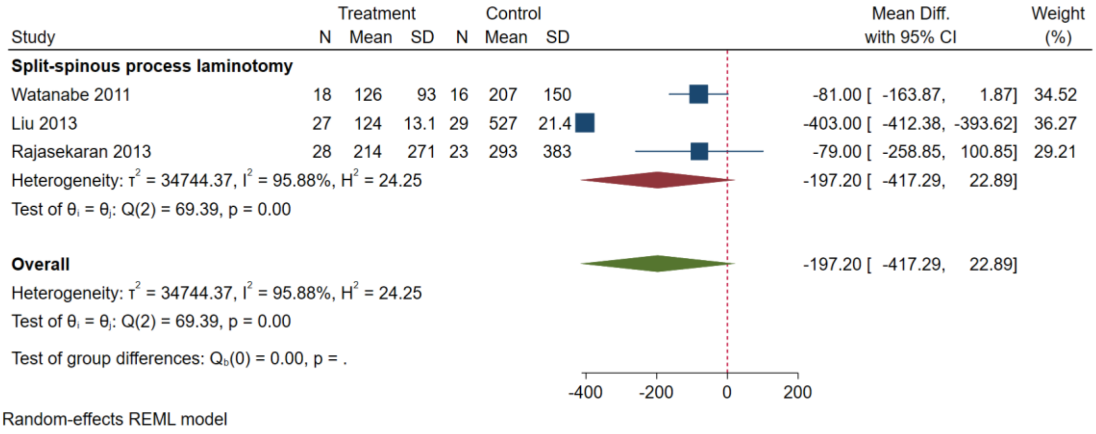

Supplementary Figure 5. Forest plot of comparison: posterior technique compared with conventional laminectomy regarding muscle atrophy ratio of paravertebral muscle.

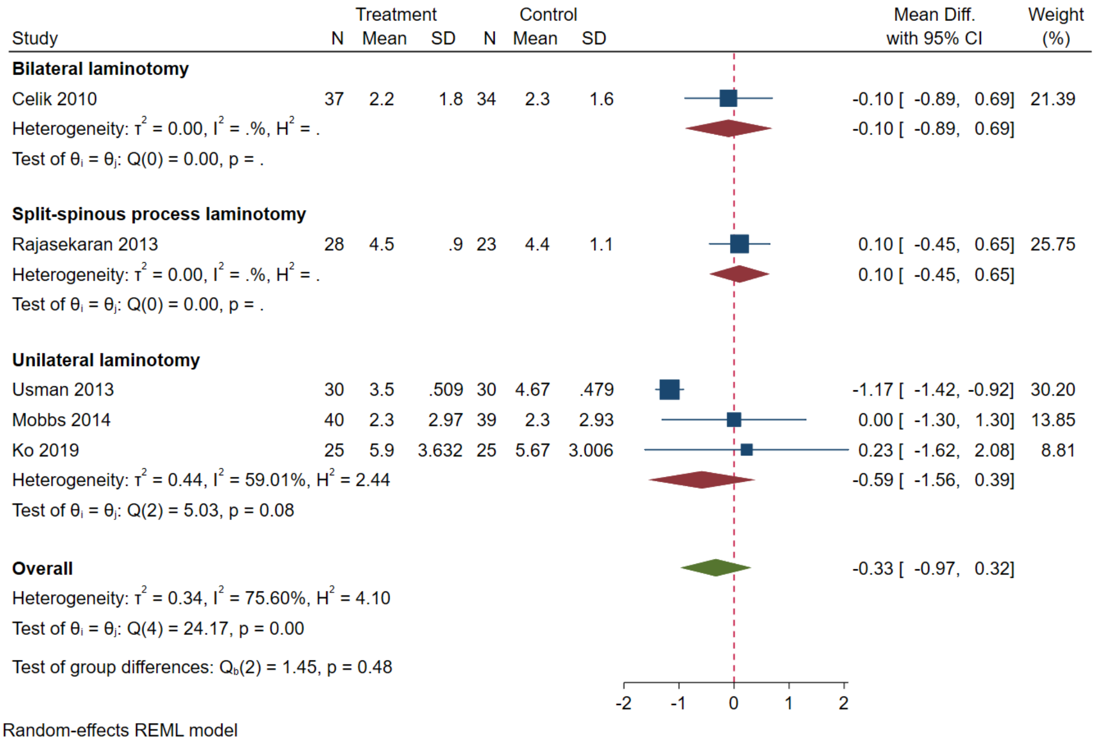

Supplementary Table 1. Search strategy

Database	Search strategy
Pubmed	#1 ((low back) OR (lumbar)) OR (canal)<br>#2 (stenos) OR (Spinal Stenosis[MeSH Terms])<br>#3 #1 AND #2<br>#4 ((((((((((((Decompression, Surgical[MeSH Terms]) OR (laminectomy[MeSH Terms])) OR (Surgical Procedures, Minimally Invasive[MeSH Terms])) OR (osteotomy[MeSH Terms])) OR (Endoscopy[MeSH Terms]))) OR (decompress)) OR (laminectom)) OR (laminotom)) OR (osteotom)) OR (enlargement)) OR (minimally invasive)) OR (endoscop)<br>#5 #3 and #4<br>#6 #5 AND randomized controlled trial (pub)
Embase	#1 ((low back) OR (lumbar)) OR (canal)<br>#2 (stenos*.mp) AND (vertebral canal stenosis)<br>#3 #1 AND #2<br>#4 (decompression surgery) OR (nerve decompression) OR (laminectomy) OR (minimally invasive surgery) OR (osteotomy) OR (endoscopic surgery) OR (decompress*.mp) OR (laminectom*.mp) OR (laminotom*.mp) OR (osteotom*.mp) OR (enlargement.mp) OR (minimally invasive.mp) OR (endoscop*.mp)<br>#5 #3 AND #4<br>#6 #5 AND [randomized controlled trial]/lim
Cochrane library	#1 ((low back) OR (lumbar)) OR (canal)<br>#2 (stenos) OR MeSH descriptor: [Spinal Stenosis] explode all trees<br>#3 #1 AND #2<br>#4 MeSH descriptor: [Decompression, Surgical] this term only AND MeSH descriptor: [Laminectomy] this term only AND MeSH descriptor: [Surgical Procedures, Minimally Invasive] this term only AND MeSH descriptor: [Osteotomy] this term only AND MeSH descriptor: [Endoscopy] this term only AND decompress AND laminectomy AND laminotom AND osteotom AND enlargement AND minimally invasive AND endoscop<br>#5 #3 AND #4<br>#6 #5 in Trials

Supplementary Table 2. Inclusion/exclusion criteria of literature

PICOS	Inclusion	Exclusion
P	Patients with symptomatic degenerative lumbar stenosis.	Cases of congenital lumbar stenosis (e.g. achondroplasia) or acquired lumbar stenosis due to trauma, infection or abnormal bone metabolism (e.g. Paget's disease).
I	1) posterior decompressive technique that avoids removal of posterior midline structures (spinous processes, vertebral arches, interspinous and supraspinous ligaments) or a technique involving only partial resection of the vertebral arch;<br>2) No limit on sample size.	removal of posterior midline structures (spinous processes, vertebral arches, interspinous and supraspinous ligaments).
C	Conventional facet-preserving laminectomy.	Cases of decompression through interspinous process devices or concomitant (instrumented) fusion procedures.
O	1) Primary outcomes including functional disability (e.g. Roland Disability Questionnaire, Oswestry Disability Index), perceived recovery, leg and back pain, complications.<br>2) Secondary outcome included length of hospital stay, recovery (good + excellent), instability, surgery time, perioperative blood loss, muscle cell injury (creatine kinase level), paraspinal muscle denervation/atrophy.	Relevant outcomes were missing.
S	RCT irrespective of blinding or arm.	1) Articles without peer-reviewed or unpublished;<br>2) Studies that were repeatedly published or had qualitative outcomes;<br>3) Quasi-experimental studies, crossover, and observational studies.

We included all prospective studies comparing a posterior decompressive technique that avoids removal of posterior midline structures (spinous processes, vertebral arches, interspinous and supraspinous ligaments) or a technique involving only partial resection of the vertebral arch with conventional facet-preserving laminectomy. We also included studies that describe cases requiring decompression of more than one stenotic level or a concomitant discectomy or foraminotomy.

We excluded studies involving cases of decompression through interspinous process devices or concomitant (instrumented) fusion procedures.

Supplementary Table 3. Risk of bias table.

1	Random sequence generation (selection bias)
2	Allocation concealment (selection bias)
3	Blinding of participants and personnel (performance bias)
4	Blinding of outcome assessment (detection bias)
5	Incomplete outcome data (attrition bias)
6	Selective reporting (reporting bias)
7	Other bias

eTable 4. Characteristics of the Included Trials and Participants.

Number	Study	Study design	Comparison groups	Number of participants	Age, years	Male/female	Length of follow-up	Complete follow-up	Outcomes
1	Postacchini 1993	RCT	1) Bilateral laminotomy<br>2) Conventional laminectomy	1) 26<br>2) 32	57 (43 to 79)	34/ 36	3.7 years	67/ 70	Recovery, VAS leg pain (improvement), VAS back pain (improvement), operation duration, blood loss.
2	Thome 2005	RCT	1) Bilateral laminotomy<br>2) Unilateral laminotomy<br>3) Conventional laminectomy	1) 37<br>2) 39<br>3) 34	1) 70±7<br>2) 67±9<br>3) 69±10	1) 20/ 20<br>2) 15/ 25<br>3) 18/ 22	15.5 months	1) 37/ 39<br>2) 39/ 40<br>3) 34/ 38	RDQ, recovery, leg pain (improvement), complications, instability, walking distance, VAS back pain (improvement), operation duration, blood loss.
3	Cho 2007	RCT	1) Split-spinous process laminotomy<br>2) Conventional laminectomy	1) 40<br>2) 30	1) 61±11<br>2) 59±15	1) 16/ 24<br>2) 15/ 15	1) 15.1 months<br>2) 14.8 months	Not specified	JOA， Length of hospital stay, complications, instability, muscle cell injury, VAS back pain, operation duration, blood loss.
4	Fu 2008	RCT	1) Bilateral laminotomy<br>2) Conventional laminectomy	1) 76<br>2) 76	1) 57 (47to 70)<br>2) 57 (45 to 73)	1) 37/ 39<br>2) 33/ 43	40.6 months	Not specified	ODI, recovery, VAS leg pain, Complications, instability, walking duration, VAS back pain.
5	Yagi 2009	RCT	1) Unilateral microendoscopic laminotomy<br>2) Conventional laminectomy	1) 20<br>2) 21	1)73.3 (63to79)<br>2)70.8 (66to73)	1) 8/ 12<br>2) 6/ 15	1) 18.8 months<br>2) 18.6 months	Not specified	JOA, Length of hospital stay, complications, instability, muscle atrophy, muscle cell injury, VAS back pain, operation duration, blood loss, analgesics.
6	Celik 2010	RCT	1) Bilateral laminotomy<br>2) Conventional laminectomy	1) 37<br>2) 34	1) 59±14<br>2) 61±13	1) 17/ 20<br>2) 16/ 18	1) 5.4 years<br>2) 5.3 years	1) 37 of 40 lost to follow-up<br>2) 34 of 40 lost to follow-up	ODI, VAS，Length of hospital stay, complications, instability, walking distance, VAS back pain, operation duration, blood loss, analgesics.
7	Watanabe 2011	RCT	1) Split-spinous process laminotomy<br>2) Conventional laminectomy	1) 18<br>2) 16	1)69±10<br>2)71±8	1) 10/ 8<br>2) 8/ 8	1 year	32/34	JOA, muscle cell injury, back muscle atrophy, blood loss, operating time, analgesics, complications.
8	Gurelik 2012	RCT	1) Unilateral laminotomy<br>2) Conventional laminectomy	1) 26<br>2) 26	1) 61±10<br>2) 58±9	1) 11/ 15<br>2) 10/ 16	9.1 months	Not specified	ODI, Complications, instability, walking distance.
9	Liu 2013	RCT	1) Split-spinous process with unilateral osteotomy and laminotomy<br>2) Conventional laminectomy	1) 27<br>2) 29	1) 59±4.7<br>2) 61±3.1	1) 15/ 12<br>2) 18/ 11	2 years	Not specified	JOA, VAS leg pain, VAS back pain, muscle atrophy, muscle cell injury, complications, instability, operation time, blood loss.
10	Rajasekaran 2013	RCT	1) Split-spinous process laminotomy<br>2) Conventional laminectomy	1) 28<br>2) 23	1) 57.3±11.2<br>2) 54.5±8.2	1) 16/ 12<br>2) 14/ 9	14.2 months	51/ 52	JOA, VAS leg pain, VAS back pain, muscle cell injury, blood loss, operating time, duration of hospital stay, complications.
11	Usman 2013	RCT	1) Unilateral laminotomy<br>2) Conventional laminectomy	1) 30<br>2) 30	73.4% between 31-50 years	1) 16/ 14<br>2) 18/ 12	3 months	1) 30/ 30<br>2) 30/ 30	Operation time, length of hospital stay.
12	Mobbs 2014	RCT	1) Conventional Laminectomy<br>2) Unilateral laminotomy	1) 40<br>2) 39	1) 66.2±8.110<br>2) 68.1±10.716	1) 1:1<br>2) 1:5	24 months	1) 27/ 40<br>2) 27/ 39	ODI, RMDQ, SF-36, Pain, Operation time, hospitalization.
13	Soliman 2019	RCT	1) Bilateral laminotomy<br>2) Conventional Laminectomy	1) 109<br>2) 109	1) 54.21±4.54<br>2) 52.88±4.19	1) 60/ 49<br>2) 56/ 53	3 years	1) 109/ 109<br>2) 107/ 109	VAS leg pain, VAS back pain, ODI, neurogenic claudication (ZCQ) scores, surgical time, blood loss.
14	Ko 2019	RCT	1) Unilateral laminotomy<br>2) Conventional Laminectomy	1) 27<br>2) 27	1) 68.08±10.716<br>2) 66.24±8.110	1) 10/ 15<br>2) 8/ 17	24 months	1) 25/ 27<br>2) 25/ 27	ODI, RMDQ, SF-36, Pain, Operation time, hospitalization.
